# External Rotator Sparing with Posterior Acetabular Fracture Surgery: Does It Change Outcome?

**DOI:** 10.1155/2014/520196

**Published:** 2014-07-06

**Authors:** Halil Ceylan, Ozgur Selek, Murat Inanir, Omer Yonga, Bahar Odabas Ozgur, Ahmet Y. Sarlak

**Affiliations:** ^1^Department of Orthopaedics and Traumatology, Akademi Hospital, 41300 Kocaeli, Turkey; ^2^Department of Orthopaedics and Traumatology, Kocaeli University School of Medicine, Umuttepe, 41380 Kocaeli, Turkey; ^3^Department of Physical Medicine and Rehabilitation, Kocaeli University School of Medicine, Umuttepe, 41380 Kocaeli, Turkey; ^4^Department of Sports Management, Kocaeli University Physical Education & Sports High School, Umuttepe, 41380 Kocaeli, Turkey

## Abstract

This study analyses the results of the treatment with external rotator sparing approach in acetabular fractures to determine whether muscle sparing has a positive impact on functional outcome. 20 patients with a mean age of 45.9 years (range: 26–64) that had been treated for displaced acetabular fractures were included in this series. Short Musculoskeletal Function Assessment (SMFA) questionnaire and hip muscle strength measurement were done at the 24-month of follow-up period. The radiographic results at the final followup were excellent in 9 hips (45%), good in 6 hips (30%), fair in 4 hips (20%), and poor in one hip (5%) according to the criteria developed by Matta. The average SMFA score for all of the patients was 18.3 (range: 0–55.4). The mean dysfunctional and bother indexes were 17.2 and 20.6, respectively. The overall muscle strength deficit was 11.8%. The greatest loss of strength was in internal rotation. In patients with better postoperative reduction quality of acetabular fracture, peak torque, and maximum work of hip flexion, extension and also internal rotation maximum work deficit were significantly lower (*P* < 0.05). Accurate initial reduction and longer postoperative muscle strengthening exercise programs seem critical to decrease postoperative hip muscle weakness after acetabular fractures.

## 1. Introduction

Open anatomical reduction and rigid internal fixation with early mobilization are the standard current treatment of displaced acetabular fractures [1–7]. The key to surgical success is anatomical reduction and stable osteosynthesis with minimal soft tissue damage [8]. The most commonly preferred Kocher-Langenbeck (K-L) approach requires disruption of external hip rotators which adds to the muscular injury that accompanies these fractures [9–12]. In an effort to improve outcome and decrease morbidity that resulted in good outcomes with minimally invasive total hip arthroplasty, there have been modifications of the K-L approach to spare external hip rotators in acetabular fractures [13–16]. The outcome of surgical treatment of acetabular fractures with special emphasis on reduction quality, Short Musculoskeletal Function Assessment (SMFA) questionnaire, and hip strength deficit have been the subject of numerous studies using the K-L approach [17, 18], but no such analysis has been made using the less invasive external rotator sparing acetabular approach.

The purpose of this study is to report our results with external rotator sparing approach in acetabular fractures and to analyze whether muscle sparing has a positive impact on functional outcome.

## 2. Patients and Methods

From December 2007 to October 2010, 35 patients with displaced acetabular fractures were treated with open reduction and internal fixation using a modified posterior approach that spares the short external rotators of the hip. All patients were operated by the senior author and the outcomes were evaluated with a fellowship-trained orthopaedic trauma surgeon. One patient died because of comorbid medical conditions during followup; 8 patients were not included because of additional lower extremity pathologies. That may affect the measurement of hip muscle strength. Six patients were excluded due to incomplete data records. The remaining 20 patients with isolated acetabular fractures were enrolled for evaluation. None of these 20 patients had anterior procedure for acetabular fracture. The design and all procedures were approved by the local ethics committee. The details of the patient demographics are given in Table 1.

### 2.1. Surgical Technique

**Figure 1:**
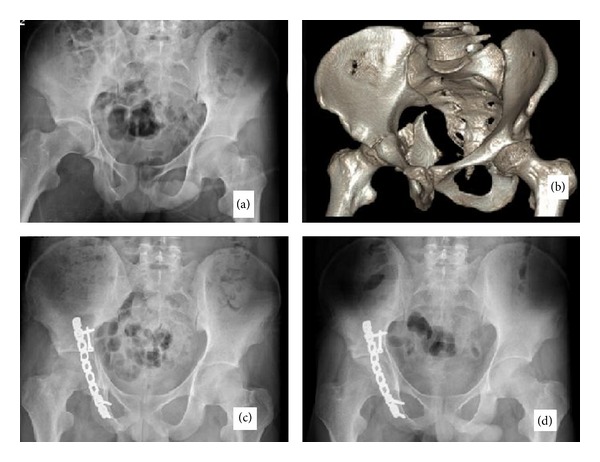
A 26-year-old male patient having acetabular fracture. (a) Preoperative X-Ray. (b) Preoperative 3D-CT scan. (c) Early postoperative X-Ray. (d) Late postoperative X-Ray (24 months).

The patients were placed in a prone position on the radiolucent normal operating table under general anesthesia. An incision beginning a handbreadth superior to posterior superior iliac spine on the iliac crest advancing laterally to the greater trochanter and then curving posteriorly towards the gluteal fold was used. Gluteus maximus origin was not detached from the iliac crest; the plane between tensor fascia lata and gluteus maximus was used to reflect the gluteus maximus posteriorly. Distal part of the gluteus maximus insertion to femur was not divided. Then gluteus maximus was reflected posteriorly to provide exposure of the entire posterior pelvis and direct visualization of the sciatic nerve. Working in the superficial plane to external hip rotators, fracture site and when necessary the joint capsule were exposed between either gluteus medius and piriformis or piriformis and superior gemellus interval (superior portal). There was short external rotator muscle damage in the patients with hip dislocation in variable degrees. But at least the quadratus femoris and gemellus inferior muscles were intact in all patients with hip dislocation. The interval between the sciatic nerve and posterior cutaneous nerve of the thigh gives direct access to ischium. Releasing the semimembranosus origin and medial retraction of biceps femoris origin, the posterolateral wall of ischium was reached. Bending template was used for plate contouring. In most fractures gentle retraction of the gluteus medius to widen the superior portal is sufficient to reduce the displaced posterior fragment gently compressing it with a periosteal elevator. A curved 3.5 mm or 4.5 mm reconstruction plate (TIPSAN stainless steel reconstruction plate system) was passed underneath the spared piriformis and short external rotators extending from lateral ischium to the inferior iliac wing compressing the fractured fragment [15]. After reduction of the fracture, fixation was achieved (Figure 1).

### 2.2. Fracture Classification and Radiological Evaluation

Preoperative and postoperative plain radiographs and CT images of the 20 patients were reviewed and classified with a fellowship-trained orthopaedic trauma surgeon. According to Letournel-Judet classification, there were 10 elementary and 10 associated type acetabular fractures [19]. These consisted of 9 posterior wall, 1 transverse, 3 posterior column-posterior wall, 4 transverse-posterior wall, and 3 T type acetabular fracture cases.

The reduction quality and radiographic results were graded according to the criteria described by Matta [5]. Follow-up reduction was assessed on anteroposterior and Judet views of the pelvis. A displacement of 1 mm or less was considered as anatomic, 1 to 3 mm as satisfactory, and greater than 3 mm as poor. The hip was graded by recent plain radiographs as excellent if the hip joint appeared normal; as good if there were mild osteophytes and no joint space narrowing; as fair if there were moderate joint space narrowing and sclerosis; as poor if there were severe loss of joint space and subchondral cysts or collapse of the femoral head. Heterotopic ossification was graded according to the criteria established by Brooker et al. [20].

Passive range of motion exercises of the hip was applied to all patients just after the operation. Isotonic (hip flexor and abductor muscle groups) and isometric (hip adductor and knee extensor muscle groups) strengthening exercises were applied. Continuous passive motion (CPM) was applied to those patients having hip joint limitation. The patients were mobilized with toe touch weight bearing using a walker or double crutches for 6 to 12 weeks.

### 2.3. Functional Assessment and Muscle Strength Testing

Short Musculoskeletal Function Assessment (SMFA) questionnaire was completed by each patient at the time of 24-month follow-up visit. The 46-item SMFA questionnaire consists of the dysfunction index, which has 34 items for the assessment of patient's functional status, and the bother index, which has 12 items for the assessment of how much patients are bothered by functional problems [17]. In the SMFA assessment system, higher scores show poorer outcome, 0 indicates normal function, and 100 reflects maximum dysfunction.

**Figure 2:**
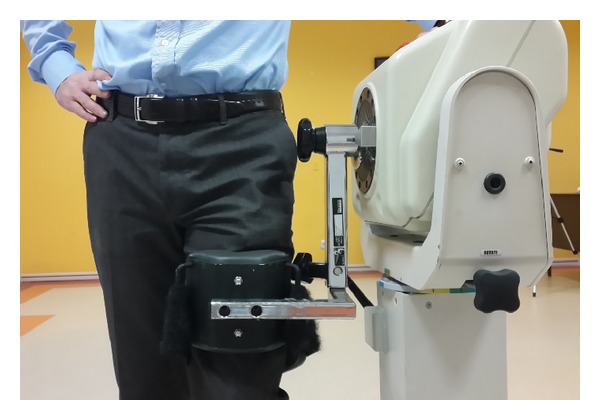
The hip muscle strength measurement by using Biodex System 3 Dynamometer. The flexion and extension muscle forces were measured at standing position.

The strength of the hip muscle groups was measured at the 24 months after acetabular fracture surgery using a Biodex System 3 Dynamometer (Biodex Medical System, Shirley, NY, USA). Dynamometer axis was calibrated according to hip rotation center for all measurements. The flexion, extension, abduction, and adduction muscle forces were measured at standing position (Figure 2); internal and external rotators were tested with the patient sitting with 90° flexion of the hip and knee joints. Five trials were performed for each injured and uninjured hip [21]. Only the trial with the best performance of peak torque (Nm) and maximum work (Joule) was used in the statistical analysis.

### 2.4. Statistical Analysis

Statistical analysis of the data obtained from the 20 patients was performed by using the chi-square and Fisher's exact tests. A *P* value of less than 0.05 was considered statistically significant (IBM SPSS Statistics for Windows, Version 20.0, IBM Corp., Armonk, NY, USA).

## 3. Results

### 3.1. Acetabular Reduction Rates

In all cases, the modified posterior approach with sparing the short external hip rotators was completely adequate to obtain fracture reduction and internal fixation. The average follow-up duration of all the patients was 35.75 months (range: 24–51 months).

The postoperative reduction was graded as anatomic in 15 hips (75%), satisfactory in 4 hips (20%), and poor in one hip (5%) (Table 2). The radiographic results at the final followup were excellent in 9 hips (45%), good in 6 hips (30%), fair in 4 hips (20%), and poor in one hip (5%) according to the criteria developed by Matta.

There was preoperative posterior hip dislocation in eight patients. Four patients developed heterotopic ossification; three of these were grade I and one was grade II. Avascular necrosis of the femoral head was not seen in any of the 20 hips. In two of the cases, superficial local wound infection that was diagnosed in the early postoperative period was treated with antibiotics without debridement. There was no iatrogenic sciatic nerve palsy postoperatively.

### 3.2. Functional Outcome and Muscle Strength Deficit

The average SMFA score for all of the patients was 18.3 (range: 0–55.4). The mean dysfunctional and bother indexes were 17.2 (range: 0–53.7) and 20.6 (range: 0–66.6), respectively. There was no relationship between SMFA scores and fracture pattern (associated and elementary). The SMFA total score and dysfunction index had a significant correlation based on reduction quality (comparing anatomic and satisfactory-poor reduction groups), but bother index did not show the same correlation. The mean SMFA score was 15.9 and the dysfunction index was 14.4 for the anatomic reduction group (*n* = 15). For the satisfactory-poor reduction group (*n* = 5), the mean SMFA score was 20.7 and the dysfunction index was 20.1. However, bother indexes for the anatomic and satisfactory-poor reduction groups were similar, with means of 19.9 and 20.5, respectively.

The overall muscle strength deficit was 11.8%. The muscle strength of the injured side was weaker than uninjured side for all patients. The greatest loss of strength was in internal rotation. According to peak torque measurement, flexion was 9.8%, extension was 8.8%, abduction was 10.9%, adduction was 12.6%, internal rotation was 15.6%, and external rotation was 13.3% weaker than the normal side. According to maximum work deficit, flexion was 10.9%, extension was 11.6%, abduction was 12.2%, adduction was 15.2%, internal rotation was 15.5%, and external rotation was 19.2% weaker compared to the normal side (Table 3).

Statistically significant differences were found in hip extension maximum work deficit (*P* = 0.019) and internal rotation peak torque deficit (*P* = 0.046) when the patients were divided into groups based on fracture pattern. These two deficits were significantly higher in associated fracture group.

In patients with better postoperative reduction quality of acetabular fracture, peak torque, and maximum work of hip flexion, extension and also internal rotation maximum work deficit were significantly lower (*P* < 0.05). For these five muscular strength measurements, patients having excellent and good results according to final radiographic grade had greater strength recovery than patients having fair and poor results (*P* < 0.05).

Flexion maximum work was the only significant parameter correlated to functional outcome. Poorer functional outcome was correlated with higher flexion maximum work deficit (*P* = 0.032). When patients were evaluated for the presence of heterotopic ossification and posterior hip dislocation, there was no significant difference for muscle strength deficit between groups.

## 4. Discussion

We sought to determine whether external rotator sparing approach changes the outcome in acetabular fracture surgery. Comparing the fractured extremity with uninjuried extremity, mean muscle deficit of 11.8% and low mean bother-dysfunction index levels in this study might support the positive influence of external rotator sparing acetabular approach on functional outcome.

Previous studies on the analysis of postoperative hip muscle weakness after acetabular fracture surgery had different number of patients, assessment methods, and data analysis. Dickinson's, Matta's, and Borelli's studies involved 17, 92, and 15 patients, respectively, using the traditional Kocher-Langenbeck (K-L) approach [22–24]. While Dickinson and Borelli used the dynamometer, Matta preferred the subjective Hoppenfeld method to assess hip muscle strength deficit. Matta reported 17% of the patients suffering from postoperative hip muscle weakness, though he did not mention the specific muscle groups affected [23]. In the study of Dickinson, the patients had a mean 18% muscle strength deficit, with abductor muscle strength deficit of 50%, compared to the uninjured side [22]. Borelli found a mean 8% muscle strength deficit with abduction strength deficit of 20% [24]. Kubota et al. found the strength of hip abduction muscle to be lower than the control group after open reduction internal fixation [25]. Our mean muscle strength deficit of 11.8% compares favorably with the study of Borelli, though our abduction weakness of 10.9% is significantly less, using the short external rotator sparing approach. In patients operated with the external rotator sparing hip approach, Josten and Trabold could not demonstrate any differences for external-internal rotator muscle strength measurements in particular [14]. An interesting finding of our study is a 15.6% internal rotation muscle strength deficit and a 12.6% adductor muscle strength deficit in our patients, which may be hard to explain.

SMFA is a valid, reliable, and responsive tool that provides subjective patient-oriented outcome data [18]. SMFA reference values for individuals who were not patients range from 0 to 85 with a mean of 12.7. While the clinically significant functional differences in SMFA scores are unknown [18], our SMFA score ranging from 0 to 55.4 with a mean of 18.3 may indicate residual dysfunction in patients operated for acetabular fractures. The reference value for acetabular fractures previously defined by Caroll et al. was 25.7 for mean bother index and 28.5 for mean dysfunction index [26]. Our mean bother index and dysfunction index of 20.6 and 17.2 are even comparable to previous literature on acetabular fractures treated by the minimal invasive percutaneous fixation [27, 28].

Previous investigators showed a relationship between postoperative muscle strength recovery and outcome in patients with acetabular fractures [22–24]. We were unable to find a statistically significant correlation between SMFA questionnaire and muscle strength deficit except for flexion maximum work deficit. Hip flexion movement is more important for daily activities; therefore flexion may be more effective on functional outcome.

Despite all of the advances in fracture care the one variable that continues to be the most important determinant of outcome is the accuracy or quality of intra-articular reduction [23]. Our study supports the close correlation between reduction quality and functional outcome attended to by other authors when the results of anatomic reduction are compared with those of satisfactory and poor reductions [22–24]. The anatomic reduction group had a mean SMFA score of 15.9 and the satisfactory-poor reduction group had a mean SMFA score of 20.7 supporting the importance of accuracy of reduction. Dysfunction index showed a correlation similar to total SMFA score with groups determined according to reduction quality. In patients with anatomical reduction, the bother index was not different from the other group, which might be due to subjectivity of the bother index questionnaire that is hard to interpret in a study with a small sample size.

To compare the effect of muscle strength with or without muscle sparing a prospective randomized design is ideal. In retrospective case series comparative muscle strength assessment should preferably be done on similar fracture pattern operated through each approach. The value of this study may be limited by its small sample size, the lack of an independent control group, and the heterogeneity of our patients in terms of fracture patterns with different degrees of initial fracture displacement. Although the findings of our study do not support the close relation of postoperative muscle strength deficit and functional outcome, our functional results are encouraging especially considering the absence of abductor muscle strength deficit with the short external rotator sparing modified posterior approach in acetabular fractures. More data is needed to substantiate the precise decreased morbidity and reduced muscle injury with this more limited approach. Accurate initial reduction of acetabular fractures and longer postoperative muscle strengthening exercise programs seem critical to decrease postoperative hip muscle weakness after acetabular fractures.

## Figures and Tables

**Table 1:** Demographic data of the patients.

Demographic data	
Number of patients	20
Male : female	16 : 4
Average age	45.9 years (range: 26–64)
Average followup	35.75 months (range: 24–51)
Mechanism Posterior hip dislocation	Traffic accident: 17, falling: 3 8

**Table 2:** Reduction quality according to fracture type.

Fracture type	Reduction quality		
	Anatomic	Satisfactory	Poor
Posterior wall (*n*: 9)	9		
Transverse (*n*: 1)	1		
T type (*n*: 2)	2		
Posterior colon + wall (*n*: 3)	1	1	1
Transverse + posterior wall (*n*: 4)	1	3	
Both column (*n*: 1)	1		

Total % (*n*: 20)	15 (75%)	4 (20%)	1 (5%)

**Table 3:** The peak torque and maximum work deficit range for hip movement.

	Flexion	Extension	Abduction	Adduction	Internal rotation	External rotation
Deficit range (%) (peak torque)	9.8	8.8	10.9	12.6	15.6	13.3
Deficit range (%) (maximum work)	10.9	11.6	12.2	15.2	15.5	19.2
